# Supplementary material for: Application of Neuromuscular Blockers in Patients with ARDS in ICU: A Retrospective Study Based on the MIMIC-III Database
Source: J Clin Med. 2023 Feb 27;12(5):1878. doi: 10.3390/jcm12051878 (PMC10003530; doi:10.3390/jcm12051878)
Supplement: Supplementary file 1 [file jcm-12-01878-s001.zip › jcm-2134366-supplementary.pdf]

Table S1: Univariate COX analysis of 28-day mortality

Characteristics	HR	95%CI	P value
Gender			
Female	Ref	Ref	
Male	0.98	0.70 - 1.37	0.91
Age	2.35	1.10 - 5.02	0.03
Admission type			
Elective	Ref	Ref	
Emergency	1.73	0.76 - 3.91	0.19
Urgent	1.23	0.37 - 4.02	0.74
Ethnicity			
Black	Ref	Ref	
White	0.79	0.38 - 1.64	0.53
Hispanic	0.62	0.16 - 2.34	0.48
Other	2.21	1.06 - 4.60	0.04
SAPSII	2.54	1.80 - 3.58	<0.01
SOFA	3.21	2.25 - 4.58	<0.01
ARDS severity (severe vs moderate)	1.49	1.07 - 2.07	0.02
RRT firstday	1.04	0.53 - 2.04	0.91
Ventilation firstday	1.14	0.68 - 1.92	0.62
Vasopressors firstday	1.29	0.93 - 1.80	0.13
SOFA	2.54	1.80 - 3.58	<0.01
SAPSII	3.21	2.25 - 4.58	<0.01
CHF	1.14	0.82 - 1.60	0.44
AFIB	1.12	0.77 - 1.63	0.54
Renal	1.58	0.92 - 2.69	0.10
Liver	2.12	1.26 - 3.57	<0.01
COPD	1.02	0.64 - 1.64	0.93
Stroke	0.8	0.43 - 1.48	0.48
CAD	1.12	0.71 - 1.77	0.62
Malignancy	1.92	1.33 - 2.79	<0.01
Heart rate	0.73	0.51 - 1.03	0.08
Mean blood pressure	0.78	0.54 - 1.13	0.19
Body temperature	0.45	0.31 - 0.67	<0.01
RASS	0.78	0.59 - 1.02	0.08
Tidal volume	0.81	0.56 - 1.18	0.27
Plateau pressure	1.11	0.71 - 1.71	0.65
PEEP	0.57	0.31 - 1.06	0.07
Peak inspiratory pressure	1.23	0.84 - 1.82	0.29
Respiratory rate	1.5	1.08 - 2.09	0.02
P/F ratio	0.54	0.17 - 1.69	0.29
Fluid input	0.86	0.32 - 2.33	0.77
Urine output	2.55	0.81 - 8.02	0.11

Abbreviation: P/F, PaO2/FiO2; ref, reference.

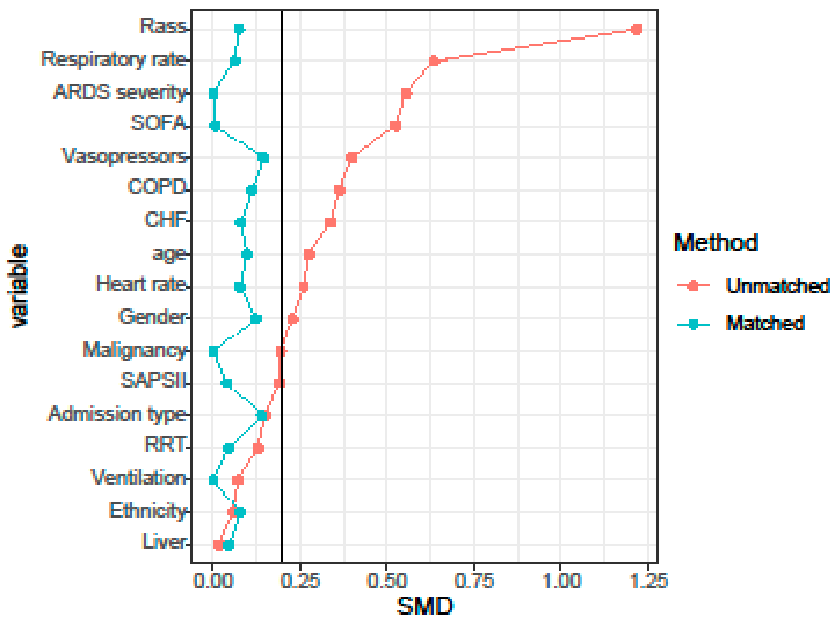

Figure S1. Standardized mean difference (SMD) of variables before and after propensity score matching.

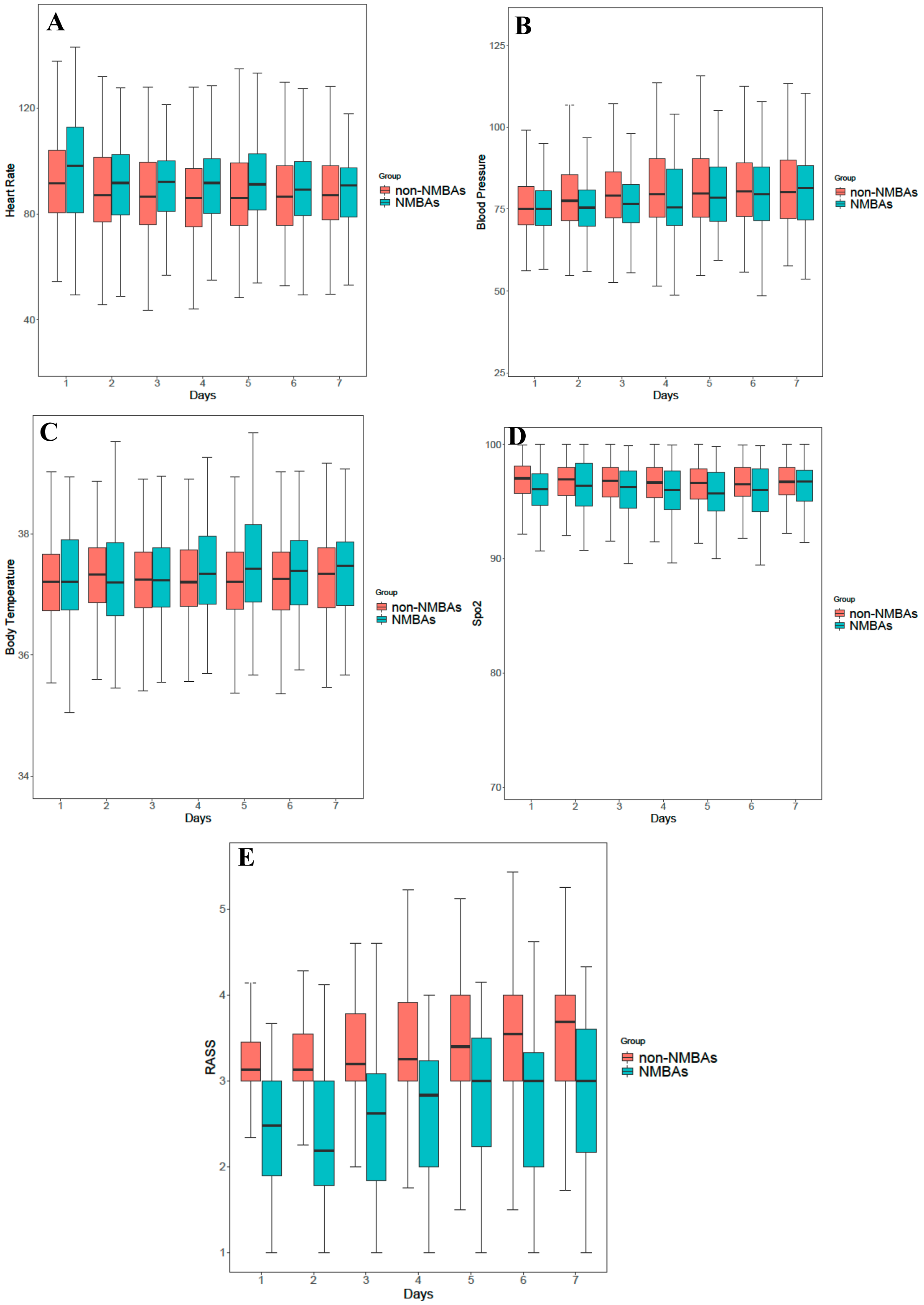

Figure S2. The vital signs of patients from the first day to seventh day. (A). Heart rate. (B). Blood pressure. (C). Body temperature. (D). SpO2. (E). RASS score.

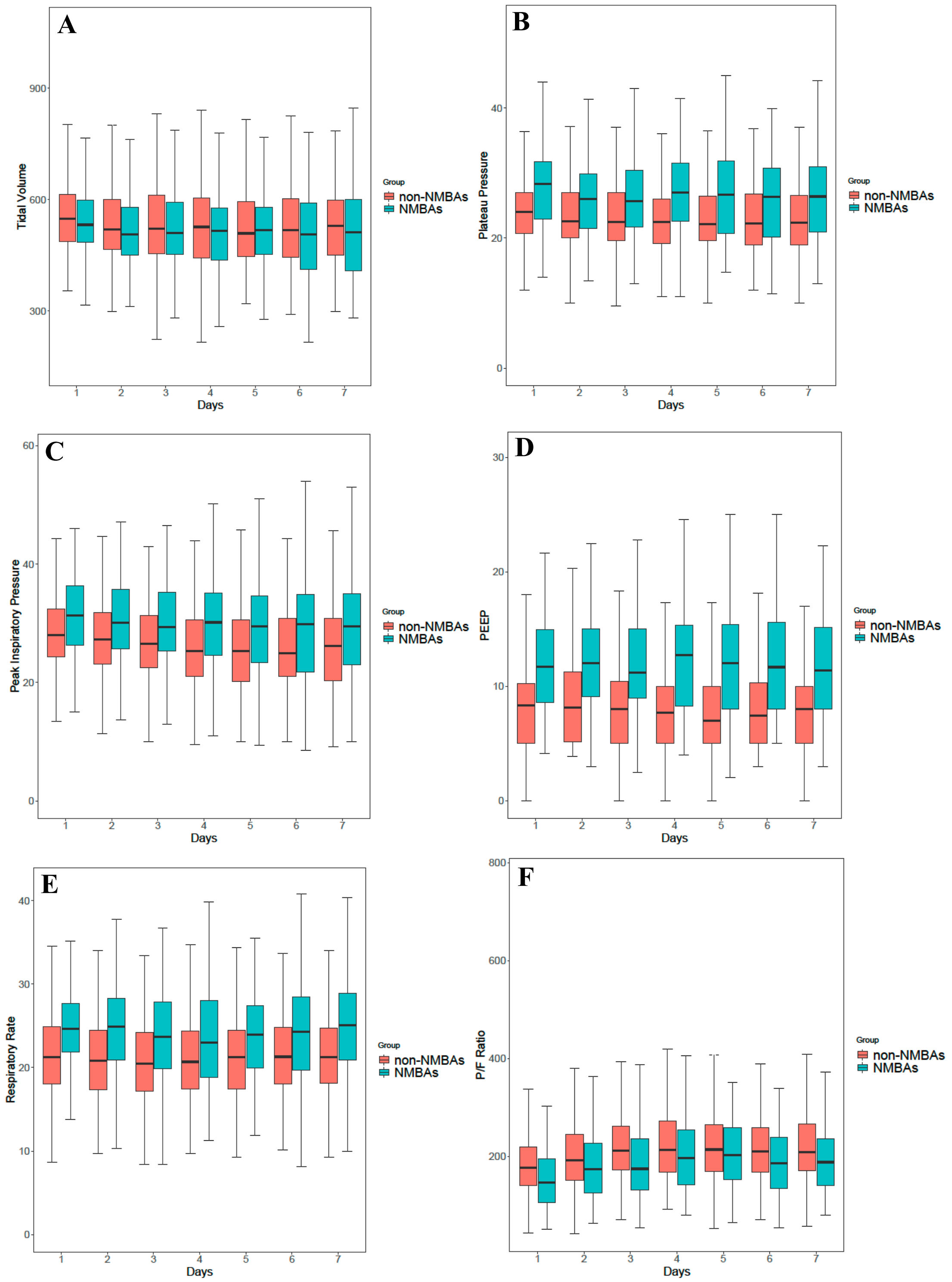

Figure S3. The respiratory mechanics indicators of patients from the first day to seventh day. (A). Tidal volume. (B). Plateau pressure. (C). Peak inspiratory pressure. (D). PEEP. (E). Respiratory rate. (F). P/F ratio.

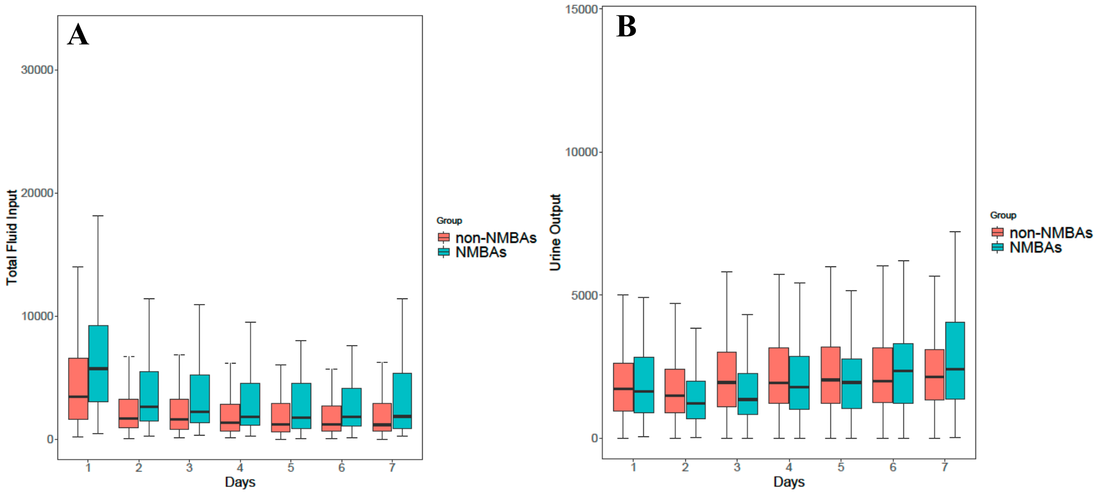

Figure S4. The total amount of fluid input and urine output of patients from the first day admitted to ICU to seventh day. (A). Fluid input. (B). Urine output.
